# Three-Year Experience With Rectal Prolapse Patients

**DOI:** 10.4021/jocmr339w

**Published:** 2010-08-18

**Authors:** Abdullah Ozgonul, Ali Uzunkoy, Ozgur Sogut, Metin Yalcin

**Affiliations:** a Department of General Surgery, Harran University, School of Medicine, Sanliurfa, Turkey; b Department of Emergency Medicine, Harran University, School of Medicine, Sanliurfa, Turkey

## Abstract

**Background:**

Rectal prolapse (RP) is a rare condition characterized by rectums protrusion through the anus with all of its layers. RP is a condition deteriorating the quality of life. Although more than 100 surgical procedures were described so far for the treatment of RP, the ideal treatment method still remains unclear. In this study, demographical data and clinical results of 13 patients who were treated at our clinic for RP for a period of 3 years were retrospectively studied, with the aim of comparing with the results of other repair methods mentioned in the literature.

**Methods:**

Total of 13 patients admitted to the general surgery unit and the emergency units between January 2008 and December 2010 were included in the study. All of the cases were treated by modified Notoras technique using various synthetic materials.

**Results:**

Of the patients, 8 were male, and 5 were female. Average age was 45.6 years (range: 23 - 79 years), and the average hospitalization time was 11.3 days (range: 3 - 19 days), with the symptom time being an average of 12 years (range: 1 - 30 years). All patients having complaints described mass prolapsing from the anal canal during defecation, rectal pain, and constipation. Six of our patients also had complaints of rectal bleeding. Average follow-up time was 24 months. No recurrence and mortality were monitored in patients who were followed.

**Conclusions:**

The main purposes in the surgical treatment of RP were to control the prolapse, and to achieve continence and remedy constipation. We believe that the modified Notoras technique made using synthetic materials the most suitable one compared to other rectopexy methods in the treatment of RP because it is safe and easily applicable.

**Keywords:**

Rectal prolapse; Rectopexy; Modified Notoras technique

## Introduction

Rectal prolapse (RP) is a rare condition characterized by rectums protrusion through the anus with all of its layers [1]. Intra-abdominal pressure increases and the contraction of the levator muscle is inhibited during defecation. And the external sphincter muscles moving in synchrony with the puborectal muscle loosens and permits the pelvic base to sediment downwards and the anorectal angle to straighten. A problem that would develop in one or more of these structures may be held responsible for a series of developments that will result in prolapse [2, 3]. Most commonly-accepted opinion about the development of rectal prolapse holds that prolapse forms with the weakening of anatomic relations with pelvic organ and tissues and also with the addition of abnormal physiological powers in intestinal habits like particularly constipation and the resulting excessive straining [4].

RP is a disease deteriorating the quality of life. It is more common in women and people over 50 years old. It is frequently associated with constipation [5]. Bleeding, fecal incontinence and rarely incarceration are observed during defecation [5, 6]. The diagnosis can be made particularly by anamnesis and anal examination in advanced level and total prolapses. Final diagnosis is made with the rectum prolapsing when patients were asked to strain at the defecation position [3, 7]. Although more than 100 surgical procedures were described so far for the treatment of RP, the ideal treatment method still remains unclear [8, 9]. Demographical data and clinical results of 13 patients who were treated at our clinic for RP for a period of 3 years were retrospectively studied, with the aim of comparing with the results of other repair methods mentioned in the literature.

## Materials and Methods

Total of 13 patients admitted to the general surgery and the emergency medicine departments between January 2008 and December 2010 were included in the study. The patients were retrospectively studied by age, sex, etiological factors, additional pathologies, hospitalization time, treatment methods, and mortality and morbidity. Following a detailed history inquiry, anoscopy and rectosigmoidoscopy as well as routine examinations were applied to the patients. Altemeier et al classification system was used to evaluate the symptoms of patients (Table 1) [10].

**Table 1:** Classification of Rectal Prolapse According to Altemeier et al [10]

Stage 1	Mucosal prolapse
Stage 2	Intusseption of the rectum or the rectisigmoid junction
Stage 3	Real rectal prolapse

After pre-operative bowel cleaning of all cases, they were treated by modified Notoras technique using various synthetic materials. In this technique, the rectum pelvic peritoneal was opened and mobilized to contain lateral ligaments. Then the polypropylene patch wound was attached, leaving the frontal wall of the rectum open, and the rectum mobilized by non-absorbable suture materials to the frontal fascia of the sacrum is pulled upwards and fixed to the patch. Thereafter the pelvic peritoneal was closed and rectopexy was completed.

## Results

Of the patients, 8 were male, and 5 were female. Average age was 45.6 years (range: 23 - 79 years), and the average hospitalization time was 11.3 days (range: 3 - 19 days), with the symptom time being an average of 12 years (range: 1 - 30 years). From the point of concomitant diseases and history of operations, 1 patient had hemorrhoid, 2 patients hade hernia operations, 1 patient had Coeliac disease and blockage of the superior mesenteric artery, 1 patient had anxiety, 3 patients had mental retardation, 1 patient had bronchitis and varicose, 1 patient had hypertension and 1 patient had myoma-induced myolysis. All of the patients complained and described mass protruding from the anal canal by defecation, rectal pain and constipation. Six of our patients also had complaints of rectal bleeding. Average follow-up time was 24 months. No recurrence and mortality were monitored in patients who were followed.

## Discussion

Proposal of so many operation methods on a disease that is surgically treated clearly reveals the difference of opinions on etiology and surgical treatment. Despite this, the ideal surgical treatment method still remains unclear. In such patients, modified rectal prolapse is the most effective treatment approach. The basic principle of rectopexy surgeries currently applied abdominally is to prevent the rectum to protrude from the anal orifice by fixing without disrupting the integrity of the rectum. One other way to do this is to create a fibrosis between the mobilized rectum and the sacrum via a synthetic substance [3, 11].

In 1963, Ripstein reported rather successful results by stitching a prosthetic material through the front of the rectum to the rectum and the sacrum for the treatment of prolapse, which he describes as a simple intusseption [8]. While the Ripstein procedure was successful in preventing prolapse with its initially described form, it initially received criticism because it could cause fecal impaction and obstruction. By time, the method was named Wells operation when Ivalon sponge was used after modification by various methods, and was named Notoras operation when polytetrafluoroethylene or polypropylene was used. Recurrence rates for rectopexy interventions applied abdominally using synthetic materials are generally reported under 5% [12]. While Morgan reported 2.6% mortality and 3.2% morbidity in his series of 150 cases, Hawley and Penfold report 4% recurrence in their series of 101 cases, with no cases of mortality [13].

In our series of 19 cases, modified Notaras technique was applied using propylene as synthetic material in all patients and no recurrence was found in any patient during their 24-month follow-ups. Whatever material is used, posterior rectopexies performed using prosthetic materials are accepted as the most successful form of prolapse treatment [9].

In conclusion, the main purposes in the surgical treatment of RP were to control the prolapse, and to achieve continence and remedy constipation. We believe that the modified Notoras technique made using synthetic materials the most suitable one compared to other rectopexy methods in the treatment of RP because it is safe and easily applicable. This study has certain restrictions like being retrospective and containing insufficient number of patients. These limitations should be taken into consideration when evaluating the outcomes of this study.
